# Supplementary material for: Instructional illustrations in children’s learning between normative and realism: An evaluation study
Source: PLoS One. 2023 Sep 15;18(9):e0291532. doi: 10.1371/journal.pone.0291532 (PMC10503736; doi:10.1371/journal.pone.0291532)
Supplement: S1 Appendix — (DOCX) [file pone.0291532.s001.docx]

**S1 Appendix**

Table A. Teachers' perceptions about employing the design standards for instructional illustrations used in teaching children in those grades instrument.

| **First Dimension: Relevance to Learning Outcomes (LO)** | | The degree of importance of the standard | | | | |
| --- | --- | --- | --- | --- | --- | --- |
| The importance of the standard relating to illustrations Instrument to learning outcomes is characterized by: | | Very  Important | Important | Moderately Important | Slightly Important | Unimportant |
| LO1 | It deals with social problems related to daily life. |  |  |  |  |  |
| LO2 | Related to the desired educational goals. |  |  |  |  |  |
| LO3 | Focuses on developing positive values and attitudes among children. |  |  |  |  |  |
| LO4 | Focuses on addressing the sub-concepts contained in the educational content of the topics. |  |  |  |  |  |
| LO5 | Present in a manner consistent with the educational texts. |  |  |  |  |  |
| LO6 | Focuses on addressing the main concepts contained in the educational content. |  |  |  |  |  |
| LO7 | Includes accompaniment post-questions (after viewing pictures and graphics). |  |  |  |  |  |
| LO8 | Contribute to the development of emotional outcomes. |  |  |  |  |  |
| **Second Dimension: Content Validity (CV)** | | The degree of importance of the standard | | | | |
| The importance of the standard of illustrations content validity (pictures and graphics) is characterized by: | | Very  Important | Important | Moderately Important | Slightly Important | Unimportant |
| CV1 | Allows the child to do hands-on activities. |  |  |  |  |  |
| CV2 | Derived from the local environment. |  |  |  |  |  |
| CV3 | Linked to the content of the intended learning. |  |  |  |  |  |
| CV4 | Explain the meaning or idea of the topic of the lesson. |  |  |  |  |  |
| CV5 | Focuses on developing a sense of patriotism in children. |  |  |  |  |  |
| CV6 | Develop the ability to observe in children. |  |  |  |  |  |
| CV7 | Develop the child's scientific thinking skills. |  |  |  |  |  |
| CV8 | Presented in a variety of life contexts |  |  |  |  |  |
| CV9 | Be colored to reflect their colors studied phenomena. |  |  |  |  |  |
| CV10 | It aligns with the content of the text |  |  |  |  |  |
| CV11 | It considers the correctness, accuracy and up-to-datedness of the information |  |  |  |  |  |
| **Third Dimension: Teaching Experiences (TE)** | | The degree of importance of the standard | | | | |
| The importance of the standard of learning experiences included in the illustrations is as follows: | | Very  Important | Important | Moderately Important | Slightly Important | Unimportant |
| TE1 | Relates to the children's past experiences. |  |  |  |  |  |
| TE2 | Help the child to build knowledge. |  |  |  |  |  |
| TE3 | Help the child to access knowledge. |  |  |  |  |  |
| TE4 | Focus on Child-centered learning. |  |  |  |  |  |
| TE5 | It enables the child to connect his previous experiences with the new ones. |  |  |  |  |  |
| TE6 | Develop children's ability on classification skill. |  |  |  |  |  |
| TE7 | Excite the questioning skill in the child |  |  |  |  |  |
| **Fourth Dimension: Learner Needs (LN)** | | The degree of importance of the standard | | | | |
| The importance of the illustrations standard to meet the child's needs is characterized by: | | Very  Important | Important | Moderately Important | Slightly Important | Unimportant |
| LN1 | Meet children's cognitive needs. |  |  |  |  |  |
| LN2 | Meet children's emotional/social needs. |  |  |  |  |  |
| LN3 | Meet children's skill needs. |  |  |  |  |  |
| LN4 | Encourage the child to self-learning. |  |  |  |  |  |
| LN5 | Consider the psychological and mental levels of children. |  |  |  |  |  |
| LN6 | Consider individual differences between children. |  |  |  |  |  |
| LN7 | Motivate children to learn. |  |  |  |  |  |
| LN8 | Children are excited to learn. |  |  |  |  |  |
