# Supplementary material for: Instructional illustrations in children’s learning between normative and realism: An evaluation study
Source: PLoS One. 2023 Sep 15;18(9):e0291532. doi: 10.1371/journal.pone.0291532 (PMC10503736; doi:10.1371/journal.pone.0291532)
Supplement: Attachment — Submitted filename: Response to Reviewers.docx [file pone.0291532.s002.docx]

**Table of Corrections**

**Reviewer 1**

| **No.** | **Comment** | **Corrections** |
| --- | --- | --- |
| 1 | In the introduction section, it is imperative for the authors to enhance their efforts in effectively emphasizing the existing gaps in the literature. By doing so, they can clearly articulate how their research aims to address and bridge these gaps, thereby contributing to the advancement of knowledge in the field. | The following paragraph has been added to the introduction  Through the analysis of preceding research [53,54,55] the significance of integrating instructional illustrations within the educational-learning process becomes evident. This is particularly crucial for young children in their early developmental stages, as their capacity to form meanings, grasp linguistic nuances, and acquire learning encounters is largely reliant on the utilization of instructional illustrations. Their learning is fundamentally grounded in tangible experiences. Consequently, prior investigations have substantiated the criticality of incorporating proficient explanations within educational materials, such as school textbooks, to effectively attain learning objectives for children. Educators emphasize that the adept incorporation of instructional illustrations is intricately tied to their skillful and fitting integration.  In Addition, Considering the preceding information, the present study stands out due to its approach towards children's books, taking into consideration the principles of effective instructional illustration design. This is particularly significant given the prevailing national focus in Jordan on developing textbooks for all children during their early years. Despite the commendable efforts made towards this objective, there remains a disparity between theoretical principles and practical application. Consequently, this study aims to bridge this gap by addressing the lack of understanding regarding male and female teachers' perspectives on the extent to which instructional illustrations comply with children's learning needs in the Jordanian context. |
| 2 | 2. In the methodology and research design section, it is imperative to provide a comprehensive definition of the population under study as well as an explanation of the sampling technique employed. Additionally, it is essential to justify the appropriateness of the chosen sampling technique and the determined sample size. | Done at section 2.2. Participants |
| 3 | 3. In order to enhance the quality and depth of the discussion section, it is crucial to incorporate a comprehensive comparison between the current findings and the existing body of literature. By engaging in such a comparative analysis, the research outcomes can be contextualized, evaluated, and potentially contribute to the advancement of knowledge in the field. | The following paragraph has been added to the section 4.2.  It could be asserted that the absence of statistically significant distinctions in teachers' responses, considering both gender and educational qualifications, may be ascribed to the circumstance that male and female educators possessing diverse academic credentials share a distinct clarity regarding the prerequisites for crafting instructional illustrations. This shared insight has facilitated instructors in effectively conveying their perspectives.  In Addition, this paragraph  These findings align with the outcomes of preceding studies that underscored the significance of training teachers in the adept utilization of instructional illustrations. Consequently, a substantial proportion of novice educators have participated in training initiatives focused on the effective deployment of instructional illustrations. Moreover, the inclusion of instructional illustration implementation as an integral facet within pre-service teacher professional development programs has been emphasized, congruent with the tenets of the constructive learning model that accentuates the proactive role of the learner. This outcome can likewise be attributed to the context in which individuals with limited teaching experience, particularly those new to the profession, embark upon their initial years of service with zeal, dynamism, and heightened motivation, particularly as they find themselves in a trial phase.  Further, this paragraph,  In light of the preceding discussion, these findings deviate from prior research [52,55,53,54] that underscores the pronounced role of instructional illustrations in enhancing the caliber of children's education. Consequently, the outcomes highlighted by the absence of clarifications are poised to exert an adverse impact on a child's psychological disposition, diminishing their curiosity and impeding their motivation to engage with and grasp cognitive subjects. This is particularly significant given that instructional illustrations serve as a potent instrument for shaping children's learning encounters in a captivating and stimulating manner. |
| 4 |  | The following paragraph has been added to the section 5. Conclusion  Based on the findings of this investigation, it is deducible that the pragmatic implications of the study underscore the imperative of directing attention towards the principles governing the design of educational and instructional illustrations. These illustrations constitute a pivotal wellspring that empowers educators to assume the role of learning activity architects and facilitators, fostering children's learning through the proficient integration of educational illustrations. Furthermore, insights drawn from the study's results affirm that the adept utilization of educational illustrations yields a substantial impact on shaping children's acquisition of knowledge, conceptual understanding, skills, values, and attitudes. Such adept utilization facilitates active learning experiences and nurtures children's accountability for their own educational journey. This resonates with the contemporary global movement emphasizing the central position of the child in the learning process. This overarching conclusion is realized by nurturing children's engagement with educational illustrations, wherein they engage in diverse scientific thought processes, thereby cultivating a deeper understanding of self and subject matter. |
| 5 | 5.Acknowledging research limitations and outlining future directions for further work is a crucial aspect of any scholarly endeavor. However, regrettably, the importance of addressing these elements has been overlooked or neglected in this particular study. Therefore, in order to rectify this oversight, it is imperative to not only recognize the limitations inherent within the research but also to shed light on potential avenues for future investigation. | Done in **5. Conclusion, Limitations, and Future directions** |
| 6 |  |  |
| 7 |  |  |
|  |  |  |
|  |  |  |

**Reviewer 2**

| **No.** | **Comment** | **Corrections** |
| --- | --- | --- |
| 1 | The empirical framework focuses on studies on the importance of illustrations and images in children's learning rather than on teachers' assessments. It is this second aspect that the study evaluates and, therefore, must be justified in more detail. In this regard, it is striking that the results of the teachers' assessment are extrapolated to the effects of the illustrations on children's learning in the discussion section. Such inferences should not take place directly as they do not share the phenomenon of inquiry. Strictly speaking, this is a study on teachers' assessment, which is important in educational terms. However, more is needed to contribute to studying the effects of illustrations or images on children's learning. | The following paragraph has been added to the introduction  Therefore, the present study stands out due to its exploration of educators' evaluations pertaining to educational and instructional illustrations, a facet of paramount significance in the advancement and revitalization of early childhood education. Unveiling the authentic essence of visual aids within children's literature by apprehending the perspectives and evaluations of early childhood educators constitutes a foundational concern in enhancing the caliber of these visual materials. This enhancement aims to empower teachers in effectively leveraging such resources within children's learning milieus. To attain a comprehensive panorama of educators' appraisals, the current investigation endeavors to discern differentiations in evaluations based on variables like gender, educational attainment, and teaching tenure. This facet assumes great import in the pursuit of educational enhancement. Undoubtedly, the adept crafting of visual aids holds the pivotal key to facilitating children's knowledge acquisition, meaning construction, and semantic development. |
| 2 | (2) The second hypothesis tested is not justified in the background, so the discussion of the findings is unclear: Why were differences by gender, age, and experience expected? Why are these differences significant for the first objective? | The following paragraph has been added to the section 4.2.  It could be asserted that the absence of statistically significant distinctions in teachers' responses, considering both gender and educational qualifications, may be ascribed to the circumstance that male and female educators possessing diverse academic credentials share a distinct clarity regarding the prerequisites for crafting instructional illustrations. This shared insight has facilitated instructors in effectively conveying their perspectives. |
| 3 |  |  |
